# Phenotypic responses of barley to *Akanthomyces muscarius* inoculation are maintained in the offspring generation

**DOI:** 10.1186/s12870-026-09569-5

**Published:** 2026-07-21

**Authors:** Krzysztof Stawrakakis, Wojciech Wysoczański, Marlena Lembicz, Paweł Olejniczak

**Affiliations:** 1 Adam Mickiewicz University in Poznan, Poznań, Greater Poland Poland; 2 Institute of Nature Conservation, Polish Academy of Sciences, Kraków Lesser Poland, Poland

**Keywords:** Entomopathogenic fungus, Crop improvement, Trade-off, Plant mycobiome, Biological control

## Abstract

**Background:**

Plant phenotypes are influenced by interactions among genetic, environmental, and microbial factors. Entomopathogenic fungi such as *Akanthomyces muscarius* are widely used in biological control, but their direct effects on plant growth and reproduction, independent of pest suppression, remain insufficiently understood. This study aimed to evaluate the effects of *A. muscarius* inoculation on the growth and reproductive parameters of barley (*Hordeum vulgare* L.) and to determine whether these effects are maintained in the offspring generation.

**Results:**

In the primary generation, inoculation with *A. muscarius* significantly affected plant performance in a cultivar-dependent manner. In ‘Morex’, inoculated plants showed reduced height but increased aboveground biomass and reproductive parameters. In ‘Golden Promise’, inoculation increased both shoot and root biomass without affecting plant height. Even though offspring plants were not reinoculated, selected phenotypic differences associated with maternal inoculation were observed in that generation. However, no culturable *A. muscarius* was detected in offspring leaves or seeds under the applied culture conditions.

**Conclusions:**

*Akanthomyces muscarius* inoculation modified growth and reproductive parameters of barley and was associated with phenotypic differences in the offspring generation under controlled conditions. The underlying mechanisms remain unresolved and may involve intergenerational plasticity or other non-genetic maternal effects.

## Introduction

Phenotypes expressed by a plant depend on the interplay between its genes and the environment. Phenotypic plasticity may allow an organism to adjust its function in response to varying external conditions [1, 2]. For example, in the presence of a specific microbiome in plant tissues, relationships with certain microorganisms, especially those with which the plant has coevolved, may induce adaptive responses in the plant host [3]. This presumption gave rise to the development of plant microbiome engineering, an intensively developing field of agriculture [4]. The aim of this discipline is to improve crops by manipulating plant microbiomes to achieve desirable interactions with specific microorganisms (e.g. [5]).

The species composition of microorganisms that inhabit plant tissues is known to substantially affect the phenotype of the host [6, 7]. These interactions can be viewed in the framework of the plant holobiont, emphasizing the interdependence of plants and their microbiome [8]. At the soil–root interface, roots create distinct compartments (rhizosphere soil, rhizoplane and endorhizosphere) that host enriched, compositionally shifted microbial communities compared with those in bulk soil, making the root zone a global hotspot for microbiome assembly and activity [9]. Some endophytic species reduce the impact of herbivores and plant pathogens [10–12], affect plant growth by producing phytohormones [13, 14], or alleviate abiotic stress, such as drought, salinity, heavy-metal toxicity and high temperatures, through microbe-mediated mitigation mechanisms [15, 16]. Plant characteristics can be improved experimentally by introducing specific microorganisms into plant tissues. Consequently, plants may acquire traits that enhance their growth and reproduction. This procedure has already been implemented on a relatively large scale in the production of economically important crops [17]. Moreover, manipulation of a crop microbiome is considered to be an eco-friendly technique for enhancing farm production in a sustainable manner with low environmental impact resulting from the reduced use of pesticides and fertilizers.

Barley production is also affected by insect-vectored diseases, including barley yellow dwarf virus (BYDV), which is transmitted by cereal aphids and can cause substantial yield losses in cereal crops, including barley [18–21]. Control of aphid vectors often relies on chemical insecticides, but this approach has limitations related to efficacy, non-target effects and the development of insecticide resistance in aphid populations [22–25]. These limitations prompt the search for alternative or complementary biological control strategies, such as the use of entomopathogenic fungi. However, utilizing entomopathogenic fungi as potential biocontrol agents also requires evaluation of their direct effects on the host plant, independent of their ability to suppress pests.

In this study, we investigated the effect of an entomopathogenic fungus, *Akanthomyces muscarius*, on the performance of two barley (*Hordeum vulgare* L.) cultivars (a comparison of the cultivars is provided in Table 1). *Akanthomyces muscarius* and related entomopathogenic fungi are mainly studied as biological control agents against insect pests. For example, isolates of *Akanthomyces* have been tested against *Bemisia tabaci* on cucumber [26], *A. muscarius* has shown biocontrol potential against *Thaumetopoea pityocampa* and plant pathogenic fungi [27], and recent work has evaluated its activity against the scale insect *Toumeyella parvicornis* [28]. Entomopathogenic fungal strains are also increasingly considered in biological control strategies against herbivores and plant-associated pests [29–32]. For example, fungi of the genus *Akanthomyces* are regarded as bioinsecticides [33, 34]. In addition to their insecticidal activity, entomopathogenic fungi may also interact directly with plant tissues as endophytes or plant-associated microorganisms [29, 35, 36]. Root inoculation with entomopathogenic fungi has been shown to alter plant growth in sweet pepper in a strain- and cultivar-dependent manner [37]. A recent genomic and ecological overview also indicates that *A. muscarius* can occur as an endophyte or plant-associated fungus in several woody plants (e.g., on maple, pine, oak, laurel, and myrtle) or on crops (e.g., cucumber, sour cherry, and cabbage) [35, 36]. In our work, the strain used was isolated from barley leaves. The choice of *A. muscarius* for this experiment was therefore based on the origin of the strain and on its known entomopathogenic potential. We used a barley-derived isolate to test whether controlled inoculation with this entomopathogenic fungus can directly affect barley growth and reproductive parameters in the absence of insect pests.

**Table 1:** Comparison of selected characteristics of the investigated barley cultivars

Trait	Factor	Morex	Golden Promise	Based on
Type	-	Spring barley	Spring barley	
Spike morphology	-	Six-rowed	Two-rowed	
Plant height	-	~ 60–107 cm		[38]
Production of biologically active compounds	indole‑3‑acetic acid	lower	higher	[39]
	Salicylic acid	higher	lower	
	Abscisic acid	higher	lower	
	Cytokinins	lower	higher	
	Gramine	none	none	[40, 41]
Resistance to important fungal pathogens	*Puccinia graminis* f. sp. avenae	resistant	susceptible	[42]
	*Pyrenophora teres* f. *maculata*	susceptible/ no data	resistant	[43]
	*Cochliobolus sativus*	resistant	susceptible/ no data	[44]

Values and qualitative comparisons are based on published literature and are provided as background cultivar characteristics. Hormone concentrations were not measured in the present experiment

Since many microorganisms that inhabit plants affect plant performance, one could expect that pest-controlling fungi such as *Akanthomyces muscarius*, in addition to harming insects, would have direct effects on the host that do not involve reduced pest infestation or indirect effects through impacts on the mycobiota composition of the plant host [27]. Thus, our goal was not to study the potential of *A. muscarius* to protect the plant against pests but to examine the possible direct effects of the fungus on the host. We also aimed to determine whether phenotypic differences associated with inoculation persist beyond the directly treated plants. Such effects can be assessed at various stages of plant development; here, we specifically investigated whether they can be observed in the offspring generation.

Phenotypic differences in the offspring generation could arise through at least two non-exclusive routes. First, the fungus may be transmitted vertically to offspring *via* seeds. Second, maternal exposure to the fungus may induce physiological or developmental changes that influence offspring phenotype without direct fungal transmission. Patterns of phenotypic plasticity may evolve and hence enable the phenotype of an organism to be better suited to the conditions that the organism faces [45]. The positive effect of the plastic response to the environment may be additionally reinforced by the transduction of environmental signals from previous generations, which would be especially adaptive if the conditions encountered by parents and offspring correlated [46]. This is more likely in sessile organisms, such as plants. Such transgenerational plasticity has been observed in many plant species [47–50] and is related to their responses to temperature, nutrient level, water availability, salinity, herbivory, and microbial infections. Thus, if the potential effect of inoculation is to be preserved to some degree in the offspring generation, then intervals between consecutive effective applications of microorganisms may be prolonged. As a result, the desired effect of microbiome engineering could be achieved at a lower cost, and a lower intensity of artificial spread of the microorganism would reduce the possible negative side effects and would be associated with a smaller negative impact on the environment.

Although transgenerational and intergenerational plasticity have been documented in plants in response to several environmental factors, it remains unclear whether controlled inoculation with an entomopathogenic fungus can be associated with phenotypic differences in the offspring generation. The main objective of this work was therefore to assess the phenotypic response of barley to *A. muscarius* inoculation and to determine whether differences associated with maternal inoculation can also be observed in the offspring generation. Using the experimental setup of two laboratory-grown cultivars subjected to fungal inoculation, we addressed the following questions.

1. Does *Akanthomyces muscarius* inoculation affect barley performance in the absence of insect pests?
2. Does the effect of inoculation depend on barley cultivar?
3. Can effects associated with *Akanthomyces muscarius* inoculation be observed in the offspring generation?

##  Methods

### Cultivars

In this experiment, we used two barley cultivars whose genomes had been sequenced: ‘Morex’ by the International Barley Sequencing Consortium [51] and ‘Golden Promise’ by the James Hutton Institute [52]. Seeds of both cultivars were obtained from the Institute of Plant Genetics, Polish Academy of Sciences, Poznań. The use of plant material complied with institutional and national guidelines.

‘Morex’ (M) is used primarily in the USA because of its relatively high yield, tolerance to low temperatures, and especially good resistance to important pathogens [53]. ‘Golden Promise’ (GP) is mostly used for alcohol production because of its characteristic taste. Golden Promise has a 7 bp deletion in the gibberellin biosynthesis gene HvGA20ox2, which is associated with reduced plant height and altered agronomic traits [54]. The major differences between these cultivars are presented in Table 1. ‘Golden Promise’ produces higher levels of indole-3-acetic acid (IAA), which is associated with cell elongation and growth, while ‘Morex’ has higher levels of salicylic acid (SA) and abscisic acid (ABA), both of which play roles in stress responses. Neither cultivar produces gramine, a secondary metabolite involved in plant defence.

### Origin, culture, and identification of the fungus

The wild strain of *Akanthomyces muscarius* used in this study was isolated from leaves of winter barley (*Hordeum vulgare* L.) grown in the Greater Poland region (52°35’15.8”N 17°43’49.2”E). Information on the cultivar of the winter barley plant from which the fungal strain was isolated was not available. The leaves were sterilized in the following solutions—75% ethanol for 30 s, 5% NaOCl for 3.5 min, and 75% ethanol for 15 s—and subsequently rinsed in sterile distilled water. Later, the leaves were fragmented and placed on Petri dishes with potato dextrose agar (PDA) medium and an antibiotic (chloramphenicol, 50 mg/L) in a growth chamber at 25 °C. When fungal hyphae appeared, they were observed and moved to new Petri dishes to produce pure fungal cultures. Morphotypes were identified using molecular methods. Their DNA was isolated using a Quick-DNA Fungal/Bacterial Miniprep Kit (Zymo Research, USA) according to the manufacturer’s instructions and stored at − 20 °C. We used the primers ITS1F [55], ITS4, NS1, NS4, LROR and LR6 [56]. PCR followed a protocol used in another study [57]. PCR products were purified with alkaline phosphatase and exonuclease I and directly sequenced using ABI BigDye Terminator ver. 3.1 (Applied Biosystems, USA). The resulting sequences were edited using Chromas software (Technelysium Pty Ltd., South Brisbane, Australia) and submitted to GenBank, which assigned them the accession numbers OP890599.1 (ITS), PX569615 (SSU), and PX569499 (LSU) for this *A. muscarius* strain. Finally, the sequences were compared with those published in the databases of the European Molecular Biology Laboratory (EMBL) and National Center for Biotechnology Information with the use of the Basic Local Alignment Search Tool [58]. Species identification was confirmed if ≥ 98% of the sequence region was identical to the reference sequence from the databases.

### Seed preparation

This surface-sterilization protocol was designed to remove epiphytic microorganisms and minimize contamination. Seeds were drawn randomly from each container. Seeds without endophytes, originating from plants devoid of endophytes, were surface sterilized by submergence and mixing in 4.5% NaOCl for 30 min, followed by rinsing with distilled water. The seeds were placed on Petri dishes with PDA medium and chloramphenicol, five on each dish, and kept in a growth chamber at 25 °C for 10 days. For each cultivar, 250 seeds were tested using 50 Petri dishes, with five seeds placed on each dish. This procedure detects only endophytes that remain viable after surface sterilization and are able to grow on PDA under our culture conditions; therefore, the presence of nonculturable or slow-growing endophytes cannot be ruled out.

### Barley inoculation with *Akanthomyces muscarius*

The process of barley inoculation followed a modified procedure described by Latch and Christensen [59] in a laminar flow cabinet as follows: young barley seedlings were inoculated by making an incision with a sterile scalpel at the coleoptile node, and mycelium (~ 2 mm long fragment) was introduced. The mycelium originated from in vitro culture and was previously subjected to several rounds of subculturing in vitro to confirm the purity of the culture. The top layer of the culture was cut using a sterile scalpel and carefully transferred *via* a needle into the cut plant tissue. The control sample was composed of seedlings that were also incised, but instead of mycelium, sterile distilled water was used. A sample of 1 ml of the sterilized water used in this process was also placed on PDA medium to ensure that it truly contained no spores. Neither fungal nor bacterial growth was observed on the medium.

### Cultivation of barley seedlings

After inoculation, the plants were kept in an incubator at 25 °C for 7 days and then in a growth chamber under the same conditions, where they grew in pots. During this time, the plates were also assessed for contamination. The control group contained no hyphae growing on the medium, whereas the inoculated groups exhibited visible, distinctive *Akanthomyces muscarius* white mycelial growth, originating from the inoculation point and extending radially through the medium. This confirmed fungal viability, primary inoculation efficacy, and the absence of contamination. In the inoculated groups, if there was any visible mycelium with distinct morphological features from those of the genus *A. muscarius*, the plate and seedlings were disposed of and replaced by a backup replicate. The inoculated and control seedlings were transferred from Petri dishes directly to plastic pots (8 × 8 × 8 cm) in sterile soil in a laminar flow cabinet. The plants were placed in a growth chamber under controlled conditions: a day temperature of 20 °C, a night temperature of 15 °C, 250 µmol m⁻² s⁻¹ (measured at the height of the table, in multiple areas – the measurement results remained equal for every spot), a photoperiod of 16 h/8 h, and a humidity of 50%. All the pots were maintained in a single growth chamber. The groups were kept separated, and the positions of individual plants were randomized. During the experiment, at each measurement time, the plants were randomly repositioned within their respective groups. A total of 120 viable seedlings were placed in the growth chamber and divided into four groups of 30 individuals each. In the offspring generation, mortality was observed in all the groups. The final sample sizes are reported in the Results section. The generation culture lasted 120 days.

### Plant measurements

The following parameters of the inoculated and control plants were assessed: plant height (from the ground to the apical part), number of shoots, number of spikes, length of each spike, and seed production expressed as total spike length as well as belowground and aboveground dry biomass. Plant height was measured at four-day intervals during the vegetative growth phase (10 sessions in total). The final measurement was performed at harvest (day 120). Total spike length was used as a proxy for reproductive performance because it integrates both spike number and spike size and can be measured nondestructively. In the maternal generation, we additionally measured the mean seed volume as an indicator of seed size. Direct counts of seed number and total seed mass were not feasible within the constraints of this experiment; however, spike morphology traits (such as spike length and spikelet number) and grain size/weight traits are well-established yield components and have been strongly associated with grain yield in barley in previous studies [60, 61]. At harvest, the aboveground parts were separated from the root system at the soil surface. The root system of each plant was carefully removed from the pot together with the surrounding soil and then gently cleaned to remove adhering substrate. Roots were washed with water until visible soil particles were removed and were subsequently blotted dry with paper towels. Aboveground parts, excluding spikes, and cleaned roots were dried separately at 60 °C until dry mass stabilized. The dry mass of shoots and roots was then measured to the nearest two decimal places. The drying procedure was performed using an SLW 240 SMART laboratory dryer by POL-EKO-APARATURA, and the measurements were performed using an AXIS AKA220 2018 scale.

### Detection of the fungus in leaves and seeds

The same detection protocol was applied to both the F₁ and F₂ generations. The effectiveness of seedling inoculation with the fungus was assessed as follows. From each seedling, a leaf fragment was collected, surface-sterilized (75% ethanol for 30 s, 5% NaOCl for 3.5 min, and 75% ethanol for 15 s), and subsequently placed on Petri dishes with PDA medium and chloramphenicol 50 mg/L. The appearance of hyphae growing from the leaves confirmed the presence of the fungus in the plant tissues. In the case of any doubts resulting from morphological differences between in vitro cultures, the fungus was passaged and identified with molecular methods according to the procedure described above. Each leaf and seed sample was tested once in both generations. Additionally, two seeds were collected from each individual plant for reisolation of the endophyte and verification of its presence. The seeds were surface-sterilized following the same protocol as that applied to the seeds prior to sowing. After sterilization, they were placed on PDA medium and monitored for fungal growth under the same incubation conditions as those used for the leaf fragments.

### Offspring generation

**Fig. 1:**
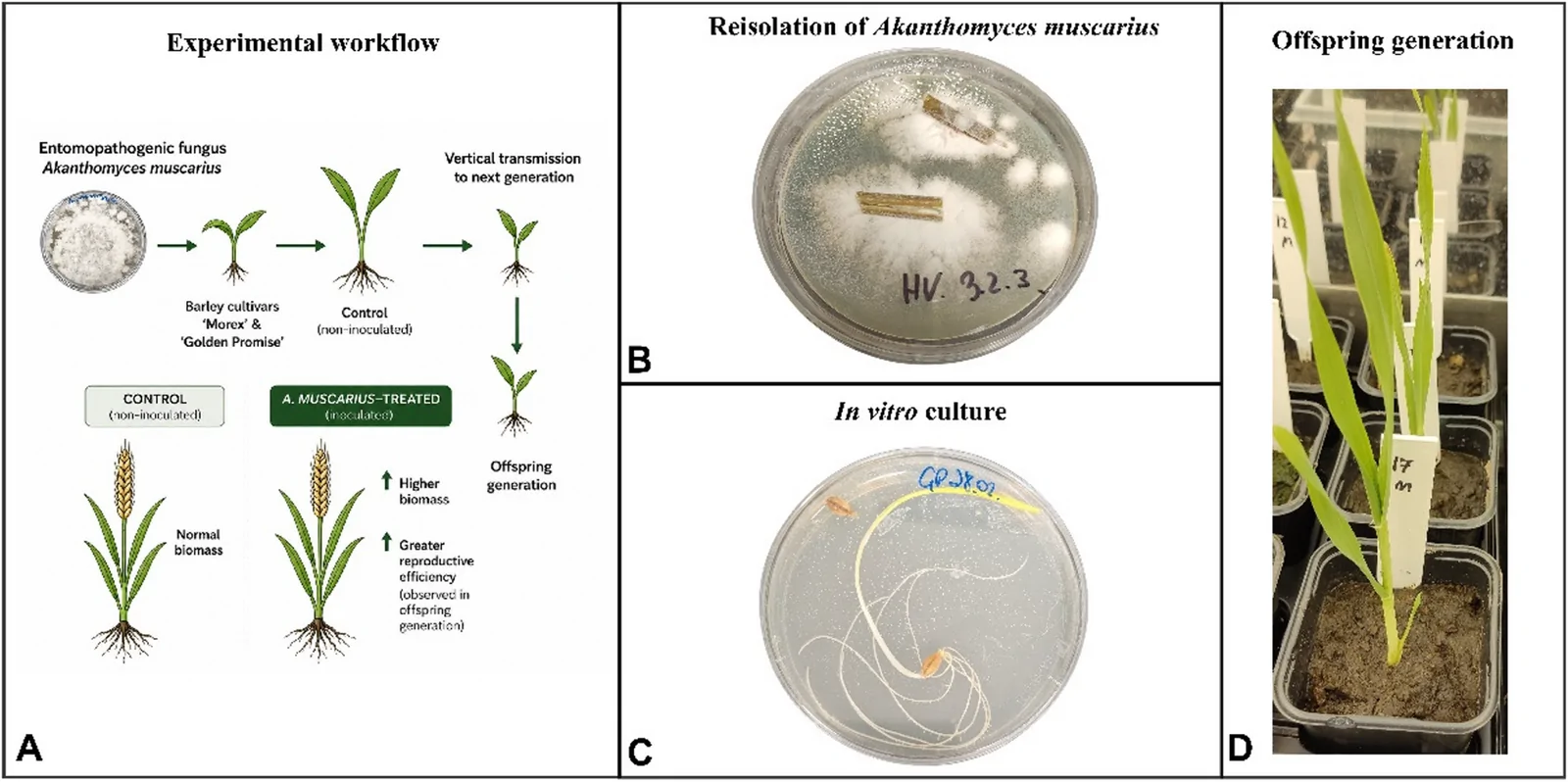
Overview of the experimental design used to evaluate the effects of *Akanthomyces muscarius* inoculation on barley and its offspring generation. **A** Schematic representation of the experiment. **B** Representative fungal re-isolation culture from plant material. **C** In vitro seedling culture used in the assessment of viable fungal mycelium. **D** Representative offspring-generation barley plant

The offspring generation of plants was obtained through self-pollination. No other barley individuals were present in the growth chamber. Seeds were collected from all F₁ plants that reached maturity and produced viable seeds. The number of F₂ individuals therefore reflected the number of surviving maternal plants. Seeds from maternal plants were collected randomly from each spike. They were then subjected to the same procedures as described in the subsection “Seed preparation”, and the seedlings were treated as in “Cultivation of barley seedlings”, except that the inoculation step was omitted. During plate cultivation, which aimed to obtain seedlings from the maternal seeds, no contamination or fungal growth originating from the seeds was observed the overall experimental workflow is shown in Fig. 1.

### Statistical analysis

The effects of inoculation (E-, E+) on both cultivars (M, GP) were analysed using two-way ANOVA. Separate analyses were conducted with biometric plant measurements included in the model as dependent variables, i.e., plant height on day 0, plant height on day 120, shoot mass, root mass, mean spike length and total spike length. These parameters were assessed for both generations of cultivated plants, and the mean seed volume was analysed only for primary generation plants. In cases where the inoculation × cultivar interaction was significant, the effect of inoculation was tested separately for the M and GP cultivars by one-way ANOVA. Differences in growth trajectories were tested by means of two-way ANOVA with inoculation and cultivar as fixed factors and with plant height assessed at four-day intervals as repeated measures. STATISTICA ver. 13.3 was used in the statistical analyses. No formal a priori power analysis was performed; sample size was determined based on growth chamber capacity and the availability of viable seedlings.

## Results

### Within-generation effects

**Fig. 2:**
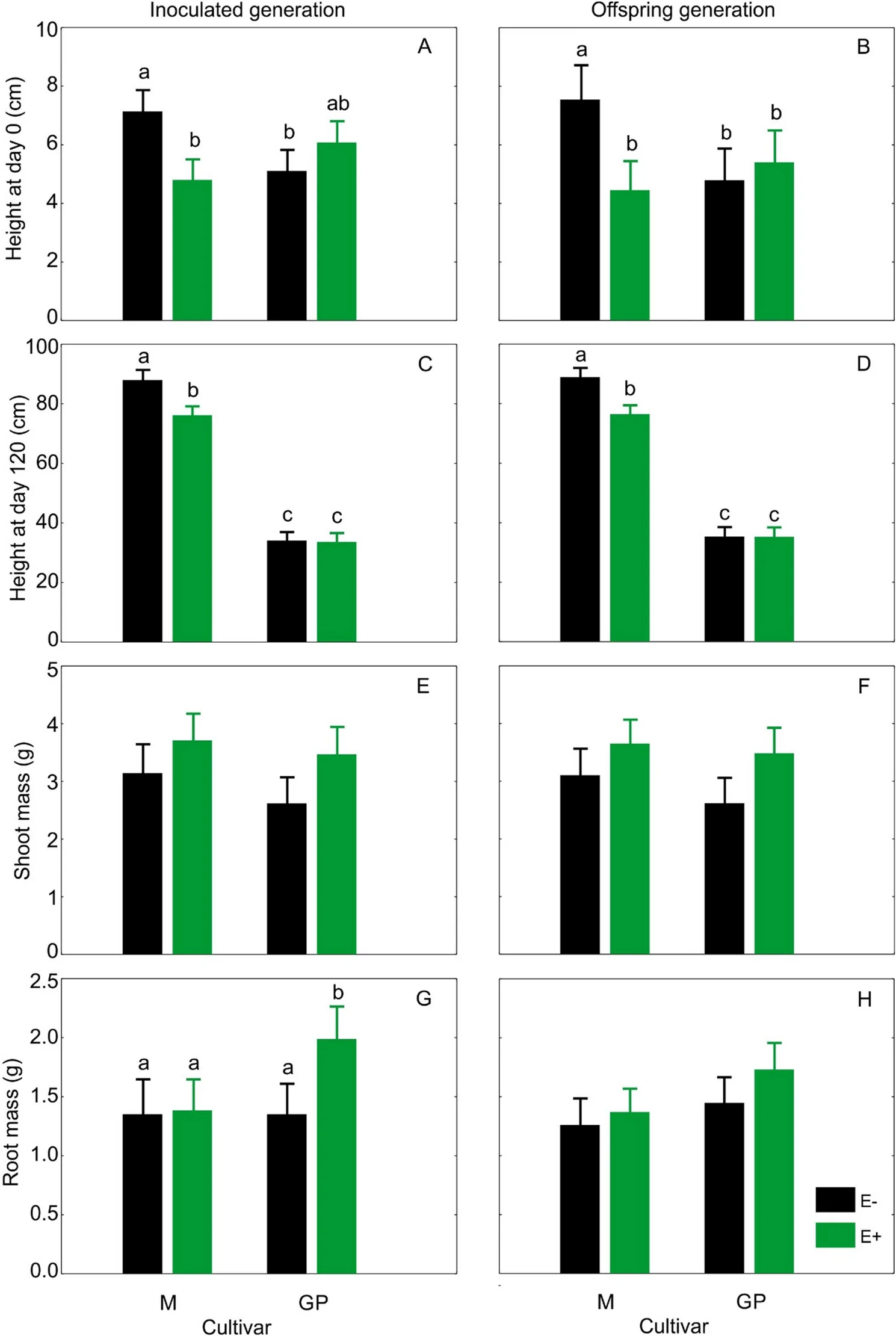
Effect of inoculation with A. muscarius (E+ inoculated plants, E- control) on plant height and biomass allocation of two barley cultivars. Mean values and 95% confidence intervals are shown for the inoculated generation (first column) and the offspring generation (second column). Panels show: **A** height at day 0 in the inoculated generation, **B** height at day 0 in the offspring generation, **C** height at day 120 in the inoculated generation, **D** height at day 120 in the offspring generation, **E** shoot mass in the inoculated generation, **F** shoot mass in the offspring generation, **G** root mass in the inoculated generation, and **H** root mass in the offspring generation. Letters above the bars indicate differences between particular groups; identical letters show non-significant differences at *p* < 0.05. When the effect of the cultivar or inoculation was significant in the two-way analysis, no letters are provided; see Tables 2 and 3 for details

**Fig. 3:**
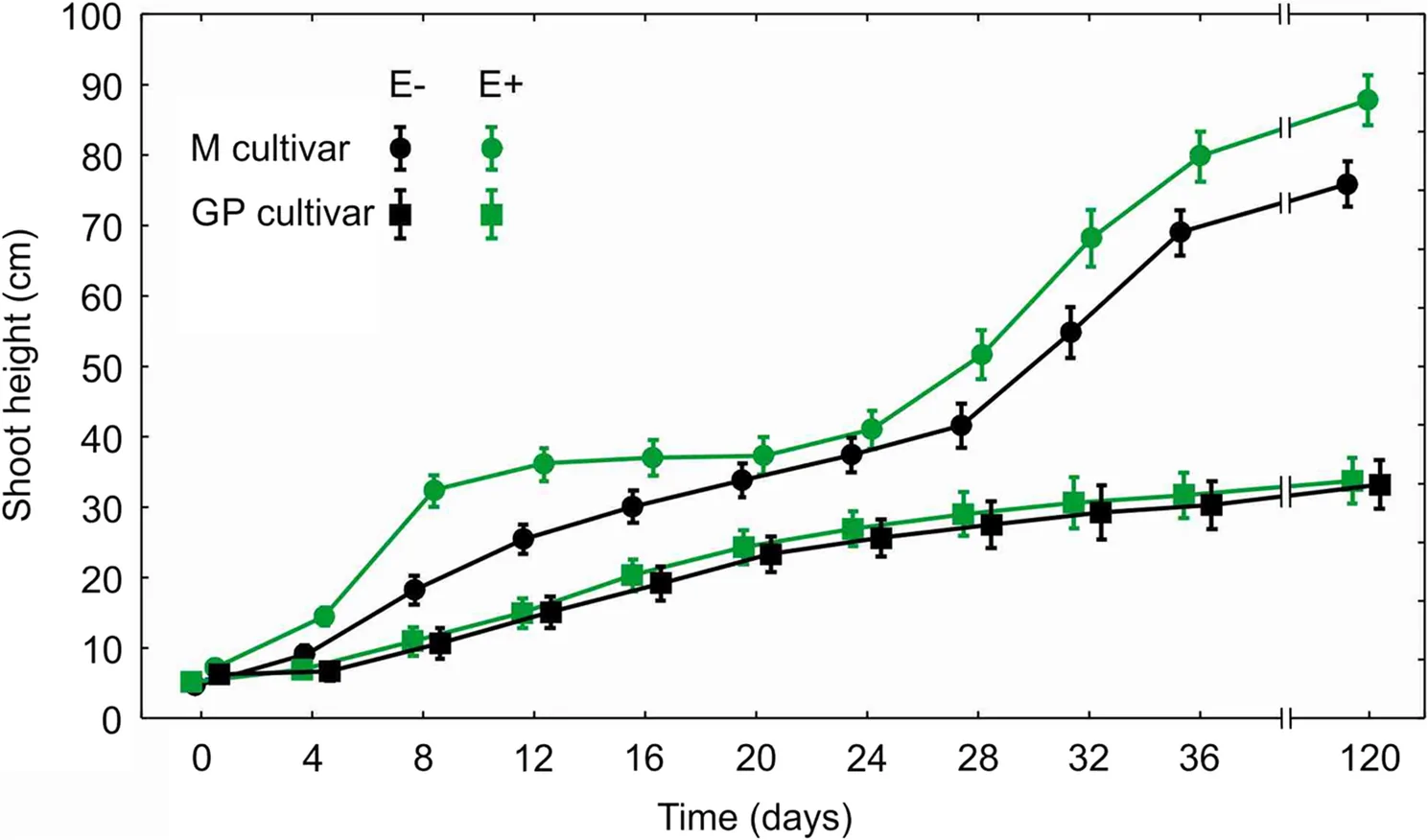
Growth curves of two barley cultivars inoculated (E+) and non-inoculated (E-) with *A. muscarius*. Mean values and 95% confidence intervals are shown

The initial number of seedlings per group was 30. In the maternal generation, the final sample sizes were as follows: control Morex (*n* = 22), inoculated Morex (*n* = 27), control Golden Promise (*n* = 27), and inoculated Golden Promise (*n* = 24). In the maternal generation, fungal reisolation was successful at an early developmental stage (after the third growth assessment), confirming effective inoculation. However, no culturable *A. muscarius* was detected at harvest (day 120) under the applied culture conditions. No fungal growth was observed in the control plants at any stage. The growth of both barley cultivars was affected by fungal inoculation, and this effect was pronounced after the initial growth of the plants in the soil. On day 0, a significant cultivar × inoculation interaction was revealed in terms of plant height (Table 2a, Fig. 2A), and one-way analysis revealed a significant height reduction in the inoculated M cultivar plants (*F*_1,58_ = 17.36; *p <* 0.001). On average, the inoculated GP plants were taller on day 0, but this effect was not significant (*F*_1,58_ = 3.96; *p =* 0.051). The growth trajectory differed between M and GP plants, and the effect of fungal inoculation was also cultivar specific (significant inoculation × cultivar interaction, *F*_1,95_ = 14.36; *p* = 0.001; Fig. 3). The final shoot height was affected differently by inoculation in the two barley cultivars (Table 2b, Fig. 2C). Compared with the control plants, the inoculated M plants were shorter on day 120 (*F*_1,47_ = 13.83; *p* = 0.001), but the inoculation did not significantly affect the final height of the GP plants (*F*_1,49_ = 0.18; *p* = 0.673).

**Table 2:** Results of ANOVA for parameters of barley assessed in the primary generation

Dependent variable	Cultivar (1)		Inoculation (2)		(1) x (2) interaction	
	F	*p*	F	*p*	F	*p*
a) Height at day 0	0.967	0.327	3.364	0.069	19.787	**< 0.001**
M cultivar			17.357	**< 0.001**		
GP cultivar			3.959	0.051		
b) Height at day 120	809.451	**< 0.001**	13.369	**< 0.001**	11.079	**0.001**
M cultivar			13.827	**0.001**		
GP cultivar			0.180	0.673		
c) Shoot mass	2.554	0.113	8.387	**0.005**	0.314	0.576
d) Root mass	4.297	**0.041**	5.435	**0.022**	4.528	**0.036**
M cultivar			0.022	0.883		
GP cultivar			9.517	**0.003**		
e) Mean spike length	0.679	0.412	0.163	0.687	0.900	0.345
f) Total spike length	71.377	**< 0.001**	12.159	**< 0.001**	2.150	0.145
g) Mean seed volume	16.322	**< 0.001**	4.873	**0.030**	0.019	0.890

First, two-way analysis was performed. When the cultivar × inoculation interaction was significant, the effect of inoculation was tested separately for the Morex (M) and Golden Promise (GP) cultivars. In all analyses, the significance for the intercept was *p* < 0.0001. Significant effects are boldfaced

**Fig. 4:**
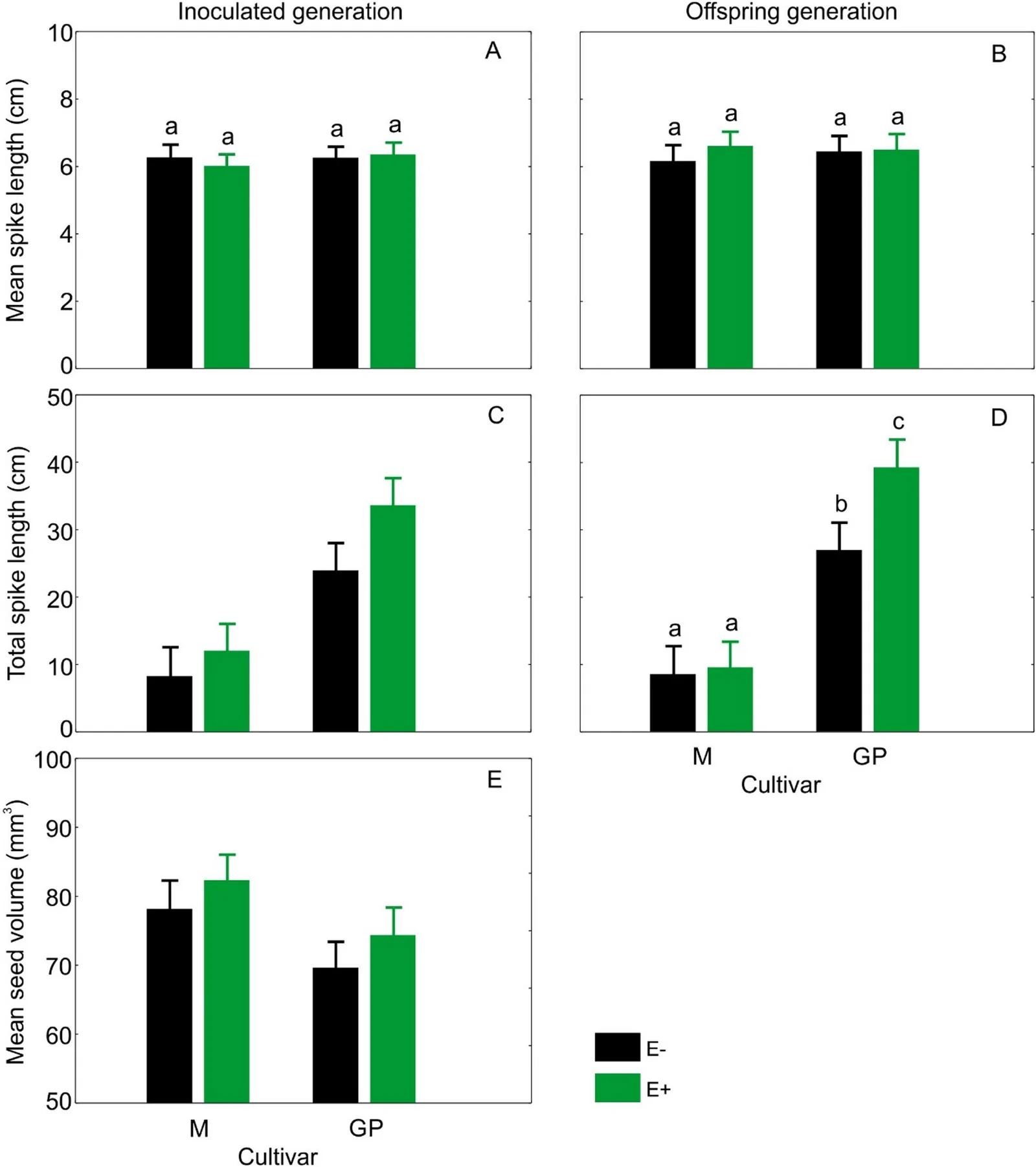
Effect of inoculation with A. muscarius (E+ inoculated plants, E- control) on reproductive parameters of two barley cultivars. Mean values and 95% confidence intervals are shown for the inoculated generation (first column) and the offspring generation (second column). Panels show: **A** mean spike length in the inoculated generation, **B** mean spike length in the offspring generation, **C** total spike length in the inoculated generation, **D** total spike length in the offspring generation, and **E** mean seed volume in the inoculated generation. Letters above the bars indicate differences between particular groups; identical letters show non-significant differences at *p* < 0.05. When the effect of the cultivar or inoculation was significant in the two-way analysis, no letters are provided; see Tables 2 and 3 for details

All the plants that survived until day 120 were harvested, and their dry mass and reproductive parameters were assessed. The inoculated plants of both M and GP had higher aboveground dry biomass (excluding spikes), and there were no differences between the cultivars (Table 2c, Fig. 2E). The belowground dry biomass responded specifically to the inoculation (Table 2d, Fig. 2G), as it was higher in inoculated GP plants than in control plants (*F*_1,49_ = 9.52; *p* = 0.003), but the experimental treatment had no effect on M plant root biomass (*F*_1,47_ = 0.02; *p* = 0.883). Mean spike length was not dependent on inoculation (M or GP) or genotype (Table 2e, Fig. 4A). The summed length of all spikes per plant, however, was positively associated with inoculation, and the values for GP were greater than those for M in both control and inoculated plants (Table 2f, Fig. 4C). The mean seed volume was also consistently stimulated by inoculation in both genotypes, but the mean seed volume was greater in the M plants than in the GP plants (Table 2g, Fig. 4E).

### Offspring-generation effects

In the offspring generation, the final sample sizes were as follows: control Morex (*n* = 22), inoculated Morex (*n* = 27), control Golden Promise (*n* = 23), and inoculated Golden Promise (*n* = 23). In the offspring generation, plants were not inoculated but were derived from the experimental groups of the maternal generation. No culturable *Akanthomyces muscarius* was detected in the leaves or seeds of the offspring generation under the applied culture conditions. Despite the absence of detectable fungal growth, phenotypic effects associated with maternal inoculation were still observed. Plant height on day 0 in the offspring generation was associated with maternal inoculation in a genotype-specific way (Table 3a, Fig. 2B), with M plants derived from inoculated maternal plants exhibiting reduced height (*F*_1,46_ = 16.10; *p* < 0.001), whereas the GP plants did not respond to the treatment (*F*_1,44_ = 0.636; *p* = 0.430). Similarly, plant height on day 120 was associated with maternal inoculation (Table 3b, Fig. 2D); M plants derived from inoculated maternal plants were shorter (*F*_1,46_ = 15.66; *p* < 0.001), with no effect of the treatment on GP plants (*F*_1,44_ = 0.006; *p* = 0.940). The dry mass of shoots (without spikes) at harvest (day 120) was higher in offspring derived from inoculated maternal plants in both cultivars (Table 3c, Fig. 2F), and the differences between the cultivars were not significant. The belowground dry mass did not differ between the control and inoculated plants, but compared with the control plants root dry mass was higher in GP plants than in M plants (Table 3d, Fig. 2H). The mean spike length in the offspring generation was not related to any of the analysed factors (Table 3e, Fig. 4B). However, the summed spike length per plant was associated with maternal inoculation (Table 3f, Fig. 4D). In the cultivar GP, the summed spike length per plant was higher in plants derived from inoculated maternal plants than in plants derived from control maternal plants (*F*_1,44_ = 8.56; *p* = 0.005), whereas in M, it was not related to inoculation (*F*_1,47_ = 1.14; *p* = 0.290).

**Table 3:** Results of ANOVA for barley plants representing the offspring generation

Dependent variable	Cultivar (1)		Inoculation (2)		(1) × (2) interaction	
	F	*p*	F	*p*	F	*p*
a) Height at day 0	2.767	0.100	5.239	**0.024**	11.631	**0.001**
M cultivar			16.098	**< 0.001**		
GP cultivar			0.636	0.430		
b) Height at day 120	794.475	**< 0.001**	13.274	**< 0.001**	12.862	**0.001**
M cultivar			15.657	**< 0.001**		
GP cultivar			0.006	0.940		
c) Shoot mass	2.528	0.154	9.720	**0.002**	0.453	0.503
d) Root mass	6.217	**0.014**	3.198	0.077	0.645	0.424
e) Mean spike length	0.141	0.708	1.158	0.285	0.690	0.408
f) Total spike length	130.457	**< 0.001**	10.087	**0.002**	7.127	**0.009**
M cultivar			1.144	0.290		
GP cultivar			8.558	**0.005**		

First, two-way analysis was performed. When the cultivar × inoculation interaction was significant, the effect of inoculation was tested separately for the Morex (M) and Golden Promise (GP) cultivars. In all analyses, the significance for the intercept was *p* < 0.0001. Significant effects are boldfaced

## Discussion

### Within-generation effects

Inoculation with the fungus *Akanthomyces muscarius* affected the growth and reproduction of barley, and the effects differed between the two tested cultivars. The presence of the fungus reduced the height of M plants throughout the growing period. However, even though the plants grew shorter, the dry mass of their aboveground parts at the end of the experiment was higher, with no effect observed in terms of dry root mass. Inoculated plants of this cultivar produced spikes of the same mean length as control ones did, but the summed-up length of all the spikes per plant was significantly greater than that of noninoculated plants. In contrast, the height of the GP plants was not affected by the introduced fungus. The dry aboveground mass of the inoculated GP plants of this cultivar increased, as did their root dry mass. In the cultivar GP, inoculation did not affect the mean spike length but did significantly increase the total spike length per plant. Together with the observed increase in shoot and root biomass, these results indicate that fungal inoculation did not impose detectable growth costs and may have enhanced overall resource allocation to reproductive structures. Various effects of fungi that inhabit plant host tissues have been reported for a few decades. We acknowledge that plant performance results from complex interactions within the broader microbiome; however, the present experimental design specifically addressed the phenotypic consequences of manipulating a single microbial component under controlled conditions. Plants with fungal endophytes are protected against a wide range of infections caused by bacteria, viruses and fungi, as well as against grazing by herbivores [62–68]. Fungus–plant interactions depend on many factors, such as genotype, environmental conditions, stage of development of the host plant, duration of the interactions, and many other unknown factors [65]. However, irrespective of whether fungal effects on the host are positive or negative, fungal endophytes may modify plant resource allocation to growth, reproduction and defence. Such changes can be reflected in measurable parameters such as plant height, biomass accumulation and reproductive structures. This is particularly important when fungal candidates are considered for the control of plant pathogens or pests. Before choosing a candidate biocontrol agent or bioinsecticide, it is necessary to assess its direct influence on plant growth, reproductive performance and offspring-generation responses.

In this study, parameters related to barley growth and reproductive performance changed after inoculation with the experimentally introduced fungal strain *Akanthomyces muscarius*. The changes varied between barley cultivars, and we recorded two phenotypic responses to the presence of the fungus. In cultivar M, reduced final height accompanied by increased shoot biomass suggests altered allocation patterns, possibly reflecting reduced investment in elongation growth. In contrast, the height of the cultivar GP remained similar to that of the controls, but the shoot and root biomass increased, indicating enhanced biomass accumulation without changes in vertical growth. Because plant height is widely used as a key integrative trait linking individual performance to ecosystem functioning, such shifts in allocation between height and biomass may have important agronomic and ecological consequences [69].

The response of barley to inoculation with *Akanthomyces muscarius* may depend on genotype-specific differences in physiological regulation and phenotypic plasticity. The two cultivars differ in several agronomic and physiological characteristics, including literature-based differences in hormone-related profiles summarized in Table 1. Previously reported differences in indole-3-acetic acid (IAA) and abscisic acid (ABA), which are involved in growth regulation and stress responses, may provide a useful context for interpreting the contrasting responses of ‘Morex’ and ‘Golden Promise’ to fungal inoculation [39]. However, hormone concentrations and metabolite profiles were not measured in the present study. Therefore, we cannot determine whether the observed effects were mediated by changes in IAA, ABA or other hormonal pathways. This remains an important hypothesis for future testing.

Genotype-dependent variation in barley responses has been documented under both abiotic stress and microbial-interaction contexts. Heat stress applied during floret development has been shown to affect fertility, sterility and heat-shock protein expression differently among European spring barley cultivars [70]. Cultivar-specific responses to microbial symbionts have also been observed. In barley, cultivars differ in mycorrhizal nutrient acquisition following inoculation with arbuscular mycorrhizal fungi under elevated CO₂ conditions [71]. Together, these findings support the view that barley cultivars may exhibit distinct physiological, growth and allocation responses depending on both environmental conditions and microbial associations. The contrasting responses observed in the present study are consistent with previously documented genotype-dependent plasticity. The absence of negative effects on growth and reproductive parameters suggests that *A. muscarius* inoculation does not compromise host performance and may therefore be compatible with biological control strategies.

### Offspring-generation effects

Our results indicate that maternal inoculation with *Akanthomyces muscarius* was associated with phenotypic differences in the offspring generation, i.e., in plants that were not directly subjected to the treatment. Like the maternal generation, the offspring generation responded in a cultivar-specific manner. Their height differed in the same way as that in the maternal generation, both on day 0 (Fig. 2A and B) and on day 120 (Fig. 2C and D). Considerable similarities between the generations were observed in terms of aboveground biomass (Fig. 2E and F). However, the effect of fungal inoculation on root biomass was not observed in the offspring generation (Fig. 2G and H). Additionally, a positive response in reproductive performance, measured as total spike length, was observed in both cultivars and both generations, but the stimulating effect in the M cultivar was not significant. In barley, spikelet primordia abortion and ABA dynamics during spike development are key determinants of final spike length and grain number [72]. These results suggest that phenotypic differences associated with maternal inoculation can be observed in the offspring generation, which was not subjected to the treatment. The directions of the observed changes were partly consistent between generations. Thus, if such phenotypic differences prove beneficial under relevant agronomic conditions, similar offspring-generation responses may also be favourable, even though vertical transmission was not detected under the applied culture conditions.

The partial similarity between maternal and offspring phenotypic patterns after fungal inoculation is consistent with an intergenerational phenotypic response. However, because the mechanisms were not tested, these offspring-generation differences should not be interpreted as evidence of a specific transgenerational mechanism. Parental environmental effects, including maternal effects, have long been recognized as important sources of offspring phenotypic variation [73], and transgenerational phenotypic effects have been reported in sexually reproducing annual plants in response to both abiotic stress and biotic interactions such as parental competition [74, 75]. The most frequently studied environmental factors associated with such effects include stressors such as drought or salinity. The transgenerational memory of a stress factor experienced by the mother plant can allow offspring to respond faster to environmental change or even anticipate stress when parental and offspring environments are temporally correlated. In cultivated plants, offspring phenotypic responses associated with increased biomass or reproductive performance may be agronomically desirable and could contribute to improved crop performance following microbiome manipulation.

The fact that vertical transmission was not detected by the culture-based reisolation approach used in this study suggests the absence of culturable *A. muscarius* in seeds and offspring leaf tissues. However, culture-dependent methods can underestimate fungal occurrence because recovery depends on fungal viability, growth on the selected medium and incubation conditions [76]. The PDA-based method used here provides evidence for culturable viability, but it is not sufficiently sensitive to exclude low-abundance, dormant, slow-growing or non-culturable fungal cells. Molecular approaches, including qPCR ITS-based detection with strain-specific markers, would increase the sensitivity of fungal tracking. Nevertheless, DNA-based detection would also require careful interpretation because standard qPCR does not by itself distinguish viable fungal cells from DNA derived from non-viable cells [77]. Taking the above points into account, low-abundance, dormant, slow-growing or non-culturable persistence of the inoculated strain cannot be fully excluded. Molecular tracking would provide stronger evidence for assessing fungal persistence and vertical transmission. Future studies should therefore combine culture-based reisolation with strain-specific molecular detection to assess the persistence of the inoculated strain.

## Conclusion

Inoculation of barley with *Akanthomyces muscarius* resulted in cultivar-dependent changes in growth and reproductive traits in the maternal generation. Although plants of the offspring generation were not reinoculated, selected phenotypic differences associated with maternal inoculation were still observed, indicating that the effects of fungal exposure may extend beyond the directly treated generation.

Our findings represent an example of intergenerational phenotypic response, but its underlying mechanism remains unresolved. Molecular confirmation is needed to clarify whether fungal persistence contributed to the observed effects. Future studies should therefore combine phenotypic assessment with strain-specific fungal tracking and analyses of physiological, molecular and epigenetic responses. Candidate microbial inoculants should be evaluated not only for their effects on target pests, but also for their potential influence on host growth, reproduction and offspring performance under controlled and field conditions.

## Data Availability

The datasets used and/or analysed during the current study are available from the corresponding author on reasonable request.

## References

[1] Bradshaw AD. Evolutionary significance of phenotypic plasticity in plants. Adv Genet. 1965;13:115–55.

[2] Schlichting CD. Evolution of phenotypic plasticity. Annu Rev Ecol Syst. 1986;17:667–93.

[3] Bulgarelli D, Schlaeppi K, Spaepen S, Van Themaat EVL, Schulze-Lefert P. Structure of plant microbiota. Annu Rev Plant Biol. 2013;64:807–38. 10.1146/annurev-arplant-050312-120106. 23373698

[4] Hanif M, Raza W, Maqbool N, Ma X. Microbiome engineering: applications in sustainable crop production. Agriculture. 2024;14:89.

[5] Martin TN, Vey RT, Vieira FCB, Jacques RJS, Ferreira MM. Bradyrhizobium coinoculation. Symbiosis. 2023;91:119–37. 10.1007/s13199-023-00951-7.

[6] Vandenkoornhuyse P, Quaiser A, Duhamel M, Le Van A, Dufresne A. The importance of the microbiome of the plant holobiont. New Phytol. 2015;206:1196–206. 10.1111/nph.13312. 25655016

[7] Trivedi P, Leach JE, Tringe SG, Sa T, Singh BK. Plant–microbiome interactions: from community assembly to plant health. Nat Rev Microbiol. 2020;18:607–21. 10.1038/s41579-020-0412-1. 32788714

[8] Sánchez-Cañizares C, Jorrín B, Poole PS, Tkacz A. Plant holobiont. Curr Opin Microbiol. 2017;38:188–96. 10.1016/j.mib.2017.07.001. 28732267

[9] Reinhold-Hurek B, Bünger W, Burbano CS, Sabale M, Hurek T. Root microbiome. Annu Rev Phytopathol. 2015;53:403–24. 10.1146/annurev-phyto-082712-102342. 26243728

[10] Gibson CM, Hunter MS. Endosymbiotic mutualists. Ecol Lett. 2010;13:223–34. 10.1111/j.1461-0248.2009.01416.x. 20015249

[11] Mogren CL, Shikanai T. Plant–microbe interactions. Annu Rev Plant Biol. 2021;72:409–35. 10.1146/annurev-phyto-080516-035319.

[12] Bamisile BS, Dash CK, Akutse KS, Keppanan R, Afolabi OG, Hussain M, Qasim M, Wang L. Fungal endophytes: beyond herbivore management. Front Microbiol. 2018;9:544. 10.3389/fmicb.2018.00544. PMC5876286 29628919

[13] Ortíz-Castro R, Contreras-Cornejo HA, Macías-Rodríguez L, López-Bucio J. Microbial signals in plants. Plant Signal Behav. 2009;4:701–12. 10.4161/psb.4.8.9047. PMC2801380 19820333

[14] Mukherjee A, Gaurav AK, Singh S, Yadav S, Bhowmick S, Abeysinghe S, Verma JP. The bioactive potential of phytohormones: a review. Biotechnol Rep. 2022;35:e00748. 10.1016/j.btre.2022.e00748. PMC9204661 35719852

[15] Meena KK, Sorty AM, Bitla UM, Choudhary K, Gupta P, Pareek A, Singh DP, Prabha R, Sahu PK, Gupta VK, Singh HB, Krishanani KK, Minhas PS. Abiotic stress responses and microbe-mediated mitigation in plants: the omics strategies. Front Plant Sci. 2017;8:172. 10.3389/fpls.2017.00172. PMC5299014 28232845

[16] Ameen M, Mahmood A, Sahkoor A, Zia MA, Ullah MS. The role of endophytes to combat abiotic stress in plants. Plant Stress. 2024;12:100435. 10.1016/j.stress.2024.100435.

[17] dos Reis GA, Martínez-Burgos WJ, Pozzan R, Pastrana Puche Y, Ocán-Torres D, de Queiroz Fonseca Mota P, Rodrigues C, Lima Serra J, Scapini T, Karp SG, Soccol CR. Comprehensive review of microbial inoculants: agricultural applications, technology trends in patents, and regulatory frameworks. Sustainability. 2024;16:8720. 10.3390/su16198720.

[18] Henry M, George S, Arnold GM, Dedryver CA, Kendall DA, Robert Y, Smith BD. Occurrence of barley yellow dwarf virus (BYDV) isolates in different farmland habitats in western France and south-west England. Ann Appl Biol. 1993;123:315–29. 10.1111/j.1744-7348.1993.tb04094.x.

[19] Jarosova J, Chrpova J, Šíp V, Kundu JK. BYDV in cereals. Plant Pathol. 2012;61:712–22. 10.1111/j.1365-3059.2012.02644.x.

[20] Nancarrow N, Aftab M, Hollaway G, Rodoni B, Trębicki P. Yield losses from BYDV. Microorganisms. 2021;9:645. 10.3390/microorganisms9030645. PMC8003756 33808907

[21] Trzmiel K, Hasiów-Jaroszewska B. BYDV-PAS in Poland. Plants. 2023;12:3488. 10.3390/plants12193488. PMC10575233 37836229

[22] Razaq M, Atta S, Ahmed Pasha R. Insecticide effects on aphids. Int J Trop Insect Sci. 2025;45:447–55. 10.1007/s42690-024-01405-3.

[23] Bass C, Puinean AM, Zimmer CT, Denholm I, Field LM, Foster SP, Gutbrod O, Nauen R, Slater R, Williamson MS. The evolution of insecticide resistance in the peach potato aphid, Myzus persicae. Insect Biochem Mol Biol. 2014;51:41–51. 10.1016/j.ibmb.2014.05.003. 24855024

[24] Malloch G, Williamson M, Foster S, Fenton B. Grain aphid resistance report. AHDB; 2014.

[25] Malloch G, Pickup J, Highet F, Foster S, Williamson M, Fenton B. Assessment of the spread of pyrethroid resistant Sitobion avenae in the UK and an update on changes in the population structure of Myzus persicae in Scotland. Proc Crop Prot North Britain. 2016;1:223–228. https://www.cabidigitallibrary.org/doi/pdf/10.5555/20163306857

[26] Broumandnia F, Rajabpour A, Parizipour HMG, Yarahmadi F. Morphological and molecular identification of four isolates of the entomopathogenic fungal genus Akanthomyces and their effects against Bemisia tabaci on cucumber. Bull Entomol Res. 2021;111:628–36. 10.1017/S0007485321000298. 34001296

[27] Saidi A, Mebdoua S, Mecelem D, Al-Hoshani N, Sadrati N, Boufahja F, Bendif H. Dual biocontrol potential of the entomopathogenic fungus Akanthomyces muscarius against Thaumetopoea pityocampa and plant pathogenic fungi. Saudi J Biol Sci. 2023;30:103719. 10.1016/j.sjbs.2023.103719. PMC10344813 37457236

[28] Di Sora N, Turco S, Brugneti F, Isoli F, Mazzaglia A, Contarini M, Speranza S, Rossini L. The last of them: entomopathogenic effect of Akanthomyces muscarius on the scale insect pest Toumeyella parvicornis under laboratory conditions, a potential biological control candidate. Physiol Plant. 2025;177:e70533. 10.1111/ppl.70533. PMC12449707 40974186

[29] Bamisile BS, Dash CK, Akutse KS, Keppanan R, Wang L, Fombong AT. Endophytic fungal entomopathogens as biocontrol agents. Microbiol Res. 2018;217:34–50. 10.1016/j.micres.2018.08.016. 30384907

[30] Sharma I. Phytopathogenic fungi. Fungi Bio Prospects in Sustainable Agriculture, Environment and Nano Technology. Academic; 2021. pp. 155–88. 10.1016/B978-0-12-821394-0.00007-X.

[31] Wallis CM, Sisterson MS. Fungal biocontrol optimization. Front Fungal Biol. 2024;5:1443343. 10.3389/ffunb.2024.1443343. PMC11324555 39149520

[32] Wang L, Keyhani NO, Xia Y, Xie J. Entomopathogenic fungi limitations. Entomol Gen. 2024;44:4. 10.1127/entomologia/2024/2498.

[33] European Food Safety Authority (EFSA). Lecanicillium muscarium risk assessment. EFSA J. 2010;8:1635. 10.2903/j.efsa.2010.1635. PMC13129919 42077619

[34] European Food Safety Authority (EFSA). Akanthomyces muscarius risk assessment. EFSA J. 2020;18:e05999. 10.2903/j.efsa.2020.5999. PMC7448024 32874313

[35] Nicoletti R, Becchimanzi A. Endophytic fungi in plant protection. J Fungi. 2020;6:122. 10.3390/jof6040122.

[36] Turco S, Drais MI, Rossini L, Di Sora N, Brugneti F, Speranza S, Contarini M, Mazzaglia A. Genomic and pathogenic characterization of Akanthomyces muscarius isolated from living mite infesting hazelnut big buds. Genes. 2024;15:993. 10.3390/genes15080993. PMC11354060 39202354

[37] Wilberts L, Rojas-Preciado N, Jacquemyn H, Lievens B. Fungal strain and crop cultivar affect growth of sweet pepper plants after root inoculation with entomopathogenic fungi. Front Plant Sci. 2023;14:1196765. 10.3389/fpls.2023.1196765. PMC10277683 37342144

[38] Hellewell KB, Rasmusson DC, Gallo-Meagher M. Enhancing yield of semidwarf barley. Crop Sci. 2000;40:352–8. 10.2135/cropsci2000.402352x.

[39] Hisano H, Matsuura T, Mori IC, Yamane M, Sato K. Hormones and regeneration in barley. Plant Physiol Biochem. 2016;99:66–72. 10.1016/j.plaphy.2015.12.005. 26735586

[40] Leland TJ, Grumet R, Hanson AD. Gramine-free barley variants. Plant Sci. 1985;42:77–82. 10.1016/0168-9452(85)90145-1.

[41] Dias SL, Chuang L, Liu S, Seligmann B, Brendel FL, Chavez BG, Hoffie RE, Hoffie I, Kumlehn J, Bültemeier A, Wolf J, Herde M, Witte CP, D’Auria JC, Franke J. Gramine biosynthesis in barley. Science. 2024;383:1448–54. 10.1126/science.adk6112. 38547266

[42] Rostoks N, Steffenson BJ, Kleinhofs A. Barley resistance gene expression. Physiol Mol Plant Pathol. 2004;64:91–101. 10.1016/j.pmpp.2004.05.006.

[43] Clare SJ, Alhashel AF, Li M, Effertz KM, Poudel RS, Zhang J, Brueggeman RS. Hybrid susceptibility locus in barley. BMC Plant Biol. 2024;24:622. 10.1186/s12870-024-05303-1. PMC11218204 38951756

[44] Steffenson BJ, Hayes PM, Kleinhofs A. Disease resistance in barley. Theor Appl Genet. 1996;92:552–8. 10.1007/BF00224557. 24166322

[45] de Jong G. Phenotypic plasticity evolution. Am Nat. 1995;145:493–512.

[46] Agrawal AA, Laforsch C, Tollrian R. Transgenerational induction of defenses in animals and plants. Nature. 1999;401:60–3. 10.1038/43425.

[47] Herman JJ, Sultan SE. Transgenerational plasticity in plants. Front Plant Sci. 2011;2:102. 10.3389/fpls.2011.00102. PMC3355592 22639624

[48] Yang F, Yang X, Baskin JM, Baskin CC, Cao D, Huang Z. Transgenerational plasticity provides ecological diversity. Perspect Plant Ecol Evol Syst. 2015;17:201–8. 10.1016/j.ppees.2015.03.003.

[49] Li K, Chen J, Wei Q, Li Q, Lei N. Transgenerational plasticity in plants. Front Plant Sci. 2018;9:1640. 10.3389/fpls.2018.01640. PMC6246981 30487805

[50] Aycan M, Nahar L, Baslam M, Mitsui T. Transgenerational plasticity in salinity tolerance of rice. J Exp Bot. 2024;75:5037–53. 10.1093/jxb/erae211. 38727615

[51] The International Barley Genome Sequencing Consortium. A physical, genetic and functional sequence assembly of the barley genome. Nature. 2012;491:711–6. 10.1038/nature11543. 23075845

[52] Schreiber M, Mascher M, Wright J, Padmarasu S, Himmelbach A, Heavens D, Milne L, Clavijo BJ, Stein N, Waugh R. A genome assembly of the barley ‘transformation reference’ cultivar Golden Promise. G3. 2020;10:1823–7. 10.1534/g3.119.401010. PMC7263683 32241919

[53] Fetch TG, Steffenson BJ, Bockelman HE, Wesenberg DM. Spring barley accessions with dual resistance. Can J Plant Pathol. 2008;30:534–42. 10.1080/07060660809507553.

[54] Teplyakova S, Lebedeva M, Ivanova N, Horeva V, Voytsutskaya N, Kovaleva O, Potokina E. Impact of the 7-bp deletion in HvGA20ox2 gene on agronomic important traits in barley (Hordeum vulgare L). BMC Plant Biol. 2017;17(Suppl 1):181. 10.1186/s12870-017-1121-4. PMC5688404 29143605

[55] Gardes M, Bruns TD. ITS primers for fungi. Mol Ecol. 1993;2:113–8. 10.1111/j.1365-294X.1993.tb00005.x. 8180733

[56] White TJ, Bruns T, Lee S, Taylor J. Fungal rRNA sequencing. In: Innis MA, Gelfand DH, Sninsky JJ, White TJ, editors. PCR protocols: a guide to methods and applications. San Diego: Academic; 1990. pp. 315–22. 10.1016/B978-0-12-372180-8.50042-1.

[57] Węgrzyn E, Dominiak-Świgon M, Górzyńska K, Chmiel J, Świtalski K, Lembicz M. Fungal microbiota in seeds. Sydowia. 2020;72:107–14. 10.12905/0380.sydowia72-2020-0107.

[58] Altschul SF, Gish W, Miller W, Myers EW, Lipman DJ. Basic local alignment search tool. J Mol Biol. 1990;215:403–10. 10.1016/S0022-2836(05)80360-2. 2231712

[59] Latch GCM, Christensen MJ. Infection of grasses with endophytes. Ann Appl Biol. 1985;107:17–24. 10.1111/j.1744-7348.1985.tb01543.x.

[60] Arisnabarreta S, Miralles DJ. Nitrogen and radiation effects in barley. Crop Pasture Sci. 2010;61:578–87. 10.1071/CP09292.

[61] Alqudah AM, Koppolu R, Wolde GM, Graner A, Schnurbusch T. The genetic architecture of barley plant stature. Front Genet. 2016;7:117. 10.3389/fgene.2016.00117. PMC4919324 27446200

[62] Clay K. Effects of fungal endophytes on grasses. Oecologia. 1987;73:358–62. 10.1007/BF00385251. 28311516

[63] Petroski RJ, Dornbos DL Jr, Powell RG. Loline alkaloids. J Agric Food Chem. 1990;38:1716–8. 10.1021/jf00098a019.

[64] Clay K. Fungi and food of the gods. Nature. 2004;427:401–2. 10.1038/427401a. 14749809

[65] Saikkonen K, Wäli P, Helander M, Faeth SH. Endophyte–plant symbiosis. Trends Plant Sci. 2004;9:275–80. 10.1016/j.tplants.2004.04.005. 15165558

[66] Olejniczak P, Lembicz M. Grass-endophyte interactions. Oecologia. 2007;152:485–94. 10.1007/s00442-007-0660-z. 17295020

[67] Rodriguez RJ, White JF Jr, Arnold AE, Redman RS. Fungal endophytes. New Phytol. 2009;182:314–30. 10.1111/j.1469-8137.2009.02773.x. 19236579

[68] Czarnoleski M, Olejniczak P, Mikołajczak P, Lembicz M, Kozłowski J. Endophytes and herbivory protection. Evol Ecol Res. 2010;12:769–77.

[69] Quan Q, He N, Zhang R, Wang J, Luo Y, Ma F, Pan J, Wang R, Liu C, Zhang J, Wang Y, Song B, Li Z, Zhou Q, Yu G, Niu S. Plant height as an indicator for alpine carbon sequestration and ecosystem response to warming. Nat Plants. 2024;10:890–900. 10.1038/s41477-024-01705-z. PMC11208140 38755277

[70] Callens C, Fernandez-Goméz J, Tucker MR, Zhang D, Wilson ZA. Heat stress responses vary during floret development in European spring barley cultivars. Front Plant Sci. 2023;13:918730. 10.3389/fpls.2022.918730. PMC9936242 36816480

[71] Thirkell TJ, Campbell M, Driver J, Pastok D, Merry B, Field KJ. Cultivar-dependent increases in mycorrhizal nutrient acquisition by barley in response to elevated CO₂. Plants People Planet. 2021;3:553–66. 10.1002/ppp3.10174.

[72] Boussora F, Allam M, Guasmi F, Ferchichi A, Rutten T, Hansson M, Youssef HM, Börner A. Spike developmental stages and ABA role in spikelet primordia abortion contribute to the final yield in barley (Hordeum vulgare L). Bot Stud. 2019;60:13. 10.1186/s40529-019-0261-2. PMC6620232 31292768

[73] Wolf JB, Wade MJ. What are maternal effects (and what are they not)? Philos Trans R Soc Lond B Biol Sci. 2009;364:1107–15. 10.1098/rstb.2008.0238. PMC2666680 19324615

[74] Suter L, Widmer A. Phenotypic effects of salt and heat stress over three generations in Arabidopsis thaliana. PLoS ONE. 2013;8:e80819. 10.1371/journal.pone.0080819. PMC3828257 24244719

[75] Waterman R, Sultan SE. Transgenerational effects of parent plant competition on offspring development in contrasting conditions. Ecology. 2021;102:e03531. 10.1002/ecy.3531. 34496058

[76] Høyer AK, Hodkinson TR. Hidden Fungi: Combining Culture-Dependent and -Independent DNA Barcoding Reveals Inter-Plant Variation in Species Richness of Endophytic Root Fungi in Elymus repens. J Fungi. 2021;7(6):466. 10.3390/jof7060466. PMC8226481 34207673

[77] Vesper S, McKinstry C, Hartmann C, Neace M, Yoder S, Vesper A. Quantifying fungal viability in air and water samples using quantitative PCR after treatment with propidium monoazide (PMA). J Microbiol Methods. 2008;72(2):180–4. 10.1016/j.mimet.2007.11.017. 18160156

